# Fracture of the Vertebræ

**Published:** 1829-05

**Authors:** 


### FRACTURE OF THE VERTEBRÆ.

*Fracture and Dislocation of a Lumbar Vertebra; Formation of Pus in the Theca Vertebralis; curious Symptoms; Death.* (St. George's Hospital.)

John Harris, forty-seven years of age, was admitted into hospital on the 8th of March, under the care of Mr. Brodie, having just received some injury of the back, the nature of which was not clearly ascertained, in jumping to the ground from a height of fourteen feet. The man was drunk at the time this took place, and was reported to have alighted on his feet. There was a good deal of ecchymosis all over the back, and something like an unnatural convexity of the spinal column, about the last dorsal or upper lumbar vertebræ. We did not examine the case at this time, and cannot, therefore, speak to the symptoms presented; suffice it to say, that the patient complained of a great deal of pain about the back and loins.

He was ordered house physic, and next day was bled to ℥xv.; but on the 10th the pain was still severe, and, the bowels being bound, he was ordered some calomel and colocynth, and more house physic. In the evening of the 11th he was restless, and required an anodyne; and on the 12th he suffered much from the pain in the lower part of the loins. However, according to the house-surgeon's report, he passed his water freely, and the blood that had been effused about the back was becoming absorbed. Castor oil was prescribed, and, on the 13th, ten ounces more blood were taken from the arm; fomentations were applied; and salines, with four grains of Dover's powder, given thrice daily. By these means the pain was considerably relieved; but it returned with increased severity in the afternoon of the 15th, when the pulse was found to be frequent and small, the skin approaching to cold, the countenance anxious and unnaturally sallow, the abdomen somewhat tense and tender under pressure.—℞. Tinct. Opi. ♏xx. Spt. Æth. c. ʒss. Mist. Camph. ℥i. statim.

Next day, at Mr. Brodie's visit, we saw the patient for the second time since his admission, when there was a remarkable alteration in his appearance. The skin and conjunctivæ were decidedly yellow; the pulse was rapid, but totally devoid of strength; the tongue dry, and of a dusky red; the patient rambling and delirious. He complained of excessive tenderness of the back, and had very little power over the muscles of the lower extremities. The belly was swollen and tympanitic; there was some degree of pain on pressing on the right hypochondriac region; no cough, nor pain in the chest on making a full inspiration. From all that we could learn, the patient had had no rigor, nor any vomiting or nausea. From the nature of the symptoms, the peculiar delirium, the yellow tint of skin, the depression of the nervous and bodily power, the state of the belly, and pain in the region of the liver, Mr. Brodie was disposed to believe that purulent depôts were forming in some of the textures of the body, and considered the case as next to hopeless. He ordered the patient: Calomel gr. iv.; Op. gr. ss. pro pil. statim sumend. Haust. Senn. ℥iss. post horas octo, et repetend. nisi prius responderit alvus. Empl. Canthar. capiti raso.

He was very noisy and delirious in the night, and on the 17th was worse. The yellowness of the skin was subdued in a kind of cadaveric hue; the teeth were dry; the mouth surrounded by herpetic eruptions; the tongue dry and red; the pulse exceedingly rapid and small. He had much pain on pressing the right hypochondrium; and some in the right side of the chest on breathing, which was short and hurried. No vomiting, nor any distinct rigor; bowels freely opened. Brandy was given; and the patient lingered out till the morning of the 18th, when he died.

*Dissection.*—The body had an uniformly yellow tinge. On cutting down upon the spine from behind, the spinous processes of the last dorsal vertebra and first lumbar were found to be separated at their apices for a full inch or more; the ligaments between them, as well as the ligamentum subflavum, uniting the bony arches from which they sprung, being torn across. The gap thus formed was the seat of a purulent depôt, extending transversely to the joint of the right articulating processes of the vertebræ above mentioned, and even beyond them, and dipping down to the surface of the theca vertebralis, with which it was in contact. There may have been about two or three drachms of pus.

On closer investigation, it was now discovered that the first lumbar vertebra was subluxated backwards from the last dorsal, their articulating processes on the right side being completely separated, the little capsule torn, and the joint laid bare and bathed in the pus of the abscess above described. The transverse process on this side was uninjured. On the left side, the displacement of the articulating processes was less; the joint was not fairly laid open, nor in contact with the matter; but the transverse process of the lumbar vertebra was broken off. The connexions of the first and second lumbar vertebræ were little, if at all, disturbed.

The body still lying on the belly, the arches of most of the dorsal and lumbar vertebræ were removed, and the state of the parts within was examined. A little, and but little, effusion of blood had taken place on the spinal sheath in the site of the injury. On opening the sheath, no pus nor marks of inflammation were discovered on the medulla, which was equally sound in its internal structure. There was no other mischief detected in the spinal column whilst looking at it thus from behind, but the traces of considerable extravasation remained in the soft parts and subcutaneous cellular membrane throughout the dorsal and lumbar regions.

On cutting through the integuments over the sternum, for the purpose of opening the chest, some very yellow gelatinous-like

2

serum was observed upon the bone, mingled with a little dirty-looking pus. There were some adhesions, most of them to all appearance old, between the pleuræ; but the lungs were sound. There were no purulent depôts in the liver, nor any thing in that viscus that could fairly account for the extreme tenderness felt during life on pressing the hypochondrium. There was scarcely a drop of urine in the bladder, and that afforded no satisfactory indication of its state, when tested by litmus and turmeric papers.

On prosecuting the examination of the spinal column from the front, a small quantity of pus was found in the loose cellular membrane over the first lumbar vertebra and behind the liver. The spinal column was cleared of the viscera, &c. when the displacement backwards of the vertebra in question was very perceptible indeed. The body of the bone was also found to have been broken transversely across, and splintered.

The head was opened, and the membranes presented marks of inflammation. There was fluid effused between the arachnoid and pia mater, and also to a considerable extent in the cells of the latter. The vessels of the membranes were very full of blood. There was no fluid in the lateral ventricles; no appearance of inflammation at the basis.

Prior to closing the report, we should mention that both arms had (what is commonly called) "festered" after bleeding: that is, the orifice itself had not healed kindly, though nothing like phlebitis had occurred.

This is an extremely interesting case, and bears upon some questions which are much agitated at the present moment. The yellow coloration of the skin was as strong as we ever remember to have observed it, and the symptoms altogether were remarkably similar to those which attend the formation of purulent depôts in various parts and tissues of the body. In all the instances, however, of visceral deposits (for example, in the liver or the lungs,) which we have observed, there have been rigors more or less frequent, accompanied with nausea and vomiting more or less severe. In this case, as we mentioned before, there were neither these symptoms nor the visceral deposits; a valuable fact, as tending not to shake our confidence in two of the most important items in our means of diagnosis. Perhaps it may be thought by some that the inflammation of the wounds of the veins of the arm played an important part: but we have fortunately (or unfortunately) seen too many of this melancholy description of cases, to believe that inflammation of the veins is a constant, or even a very frequent, concomitant; and much less a cause.*

* Medical Gazette.

